# GWAS to Sequencing: Divergence in Study Design and Analysis

**DOI:** 10.3390/genes5020460

**Published:** 2014-05-28

**Authors:** Christopher Ryan King, Dan L. Nicolae

**Affiliations:** 1 Department of Health Studies, University of Chicago, Chicago, IL 60637, USA; E-Mail: c.ryan.king@gmail.com; 2 Departments of Medicine, Statistics, and Human Genetics, University of Chicago, Chicago, IL 60637, USA

**Keywords:** sequencing, GWAS, prediction, power of genetic association

## Abstract

The success of genome-wide association studies (GWAS) in uncovering genetic risk factors for complex traits has generated great promise for the complete data generated by sequencing. The bumpy transition from GWAS to whole-exome or whole-genome association studies (WGAS) based on sequencing investigations has highlighted important differences in analysis and interpretation. We show how the loss in power due to the allele frequency spectrum targeted by sequencing is difficult to compensate for with realistic effect sizes and point to study designs that may help. We discuss several issues in interpreting the results, including a special case of the winner's curse. Extrapolation and prediction using rare SNPs is complex, because of the selective ascertainment of SNPs in case-control studies and the low amount of information at each SNP, and naive procedures are biased under the alternative. We also discuss the challenges in tuning gene-based tests and accounting for multiple testing when genes have very different sets of SNPs. The examples we emphasize in this paper highlight the difficult road we must travel for a two-letter switch.

## 1. Introduction

The Human Genome Project has paved the way to the data revolution in complex disease genetics, by permitting the development of databases of genetic variation, such as HapMap [1], and machinery for producing genome-wide data, such as genotyping arrays and high-throughput sequencing technologies. Our understanding of the genetic risk factors for complex traits has evolved from a few loci discovered with positional cloning approaches in the 1990s to thousands of replicated associations from genome-wide association studies (GWAS), available to the public, as well as scientists in the NHGRI catalog [2]. Interest has shifted recently to discovering disease association with data from whole-genome or whole-exome sequencing studies, and so far, these have had limited success. GWAS has delivered on their early promise to speed up the search for disease genes, and there are bold predictions about what sequencing can achieve [3] on the way to the era of personalized medicine. Sequencing could offer a complete picture of genetic variation—from SNPs to Copy Number Variants (CNVs) and insertions-deletions—for the subjects in the study and for future patients and has led to successful discoveries in Mendelian diseases. So far, sequencing has had limited success for complex diseases, mostly in candidate gene studies. Whole-genome and whole-exome sequencing investigations have only demonstrated the complicated architecture of common traits, sometimes indirectly through a lack of findings in single-SNP low-frequency analyses.

The success of GWAS and of the corresponding analytical tools leads naturally to an investigation of what is different between the two strategies. The goal of this paper is to compare some of the divergent aspects of GWAS and sequencing studies with the hope of guiding future sequencing investigations. We focus on two key distinctions. First, we look at consequences that follow from investigating SNPs with low minor allele frequency (MAF), including the ability to detect novel SNPs. It is important to reiterate that GWAS analyses cover, directly (through genotyping or imputation) or indirectly (through linkage disequilibrium), most of the common variants in the studied populations. This implies that the goal of sequence-based studies is to detect association with low frequency and rare variants. Even though sequencing studies can be used to investigate high MAF SNPs, we ignore their role, since traditional genotyping is dramatically more cost effective. Furthermore, we do not discuss the fact that sequencing studies permit the investigation of structural variation, an important characteristic for diseases, such as autism, where these variants play an important role. In Section 2, we develop a simple analytical formula for the power of a burden test and use it to illustrate the factors affecting power with a contrast to GWAS and scenarios for improving them. In Section 3, we illustrate two novel problems with estimation and prediction using sequencing data. First, we show that case-control studies, which add rare SNPs into a super-SNP or test the distribution of case- and control-private SNPs, can be misleading if analyzed naively. Second, we show that the optimal prediction for previously observed and novel rare SNPs can be strikingly different.

Second, we turn to issues surrounding the use of gene-based tests. In Section 4, we discuss the difficulty of selecting and tuning gene-based test statistics and contrast this to the case in GWAS. We show the alternative hypothesis, which would recommend that a particular procedure can be quite unstable even with seemingly irrelevant details of a gene. We do not recommend a particular testing procedure, but highlight concerns guiding the tuning parameter selection. Finally, in Section 5, we highlight the sharp distinctions between multiple-testing-adjustment strategies for GWAS and gene-based tests.

## 2. Power of Sequencing *versus* GWAS

The relatively minor number of associations with rare variants seems surprising to many, but was predicted by prior knowledge on the genetics of complex phenotypes. For example, the lack of major linkage loci for diseases, like type 2 diabetes [4], suggests that there are no genes with many rare variants with very large effects. Given this lack of observed associations, it is useful to investigate the relative contributions of factors driving power. We will illustrate with a burden-style test for which an analytical power calculation is straightforward.

The goal here is not to calculate power nor to find realistic sample sizes for genetic association studies with rare variants. Existing software (e.g., [5]) can perform such calculations. Our aim is to use simple analytical calculations to gain insight into what drives power and what are possible strategies for designing optimal investigations. A comparison to GWAS will illustrate the challenges ahead of us. One important set of shared assumptions for GWAS and WGAS is that of the unconfoundedness of associations. Recent work has suggested that approaches to adjusting for population structure, which work well in GWAS, may not in WGAS [6,7,8]. However, the literature on this topic is rapidly evolving, and we will set this problem aside for purposes of discussion.

Assume a balanced design with *n* cases and *n* controls. It can be shown (see Appendix A for the assumptions used in the derivation of this) that the non-centrality parameter (NCP) for burden tests [9,10] can be approximated by:

(1) $$\sqrt{n}\frac{k_{1}}{\sqrt{k}}\frac{E_{M}}{\sqrt{V_{M}+E_{M}-E_{M}^{2}}}(\gamma -1)$$

where the test is done on a set of *k* SNPs, out of which, *k*_1_ are associated with a common odds-ratio (OR) of *γ*, and *E_M_*, *V_M_* are the mean and variance of the minor allele frequency (MAF) for the SNPs in the set. This formula works for single SNP analyses, as well, with $k/\sqrt{k}=1$ and the term about frequency replaced by the corresponding function of MAF. Note that the power of the test is approximately linear in the NCP in the interesting range of moderate values.

All the terms, except the one containing elements of the MAF distribution, are easy to calculate and interpret. The MAF term can be approximated using 1000 Genomes Project data and calculations conditional on an SNP being polymorphic in a study. For 5000 cases and 5000 controls of European descent, and filtering to SNPs with MAF < 1%, that term is close to 0.046, and the non-centrality parameter when *k* = 100, *k*_1_ = 10 and *γ* = 3 is approximately 6.47. Those settings yield a power of 87% at the genome-wide 5 × 10^−8^ significance level. We will discuss the four terms in Formula (1) and contrast the results between GWAS and sequencing.

Sample size: The simplest way to double the NCP is to increase the sample size by a factor of four. This requires the least amount of innovation, but takes a huge effort and expense, especially when using existing cohorts, since ascertaining and phenotyping additional samples comparable with existing data is very difficult. As is common with many GWAS meta-analyses, a cost-effective increase in the sample size requires the use of ancestry-diverse populations. Additional diversity increases heterogeneity and will affect power to a larger degree than in GWAS, both because the effective MAF decreases (many rare alleles are population-specific) and because a similarly defined set of SNPs (e.g., all exonic SNPs in a given gene) will have different elements in different populations, with powerful tests requiring the presence of functional/causal variants in each (sub)population. We also anticipate that cryptic gene-environment interactions (GxE) provides a substantial amount of heterogeneity in effect sizes. GxE has been long known to exist for some complex traits (e.g., for a review in psychiatric phenotypes, see [11]); given how difficult is it to anticipate relevant modifiers, measure them accurately and statistically detect them [12], it seems likely that unknown environmental modifiers are not uniformly distributed across populations. We will not expand on the difficulties inherent to adjusting for structure in diverse samples, but note that this is much more challenging in sequencing, since rare SNPs can be specific for relatively recent and small-scale demographic events [9,10].

**Figure 1:**
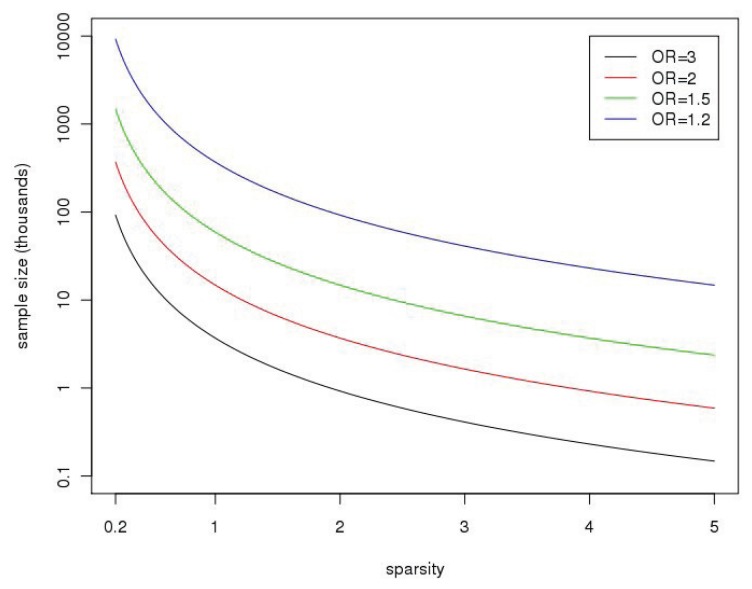
The plot shows the sample sizes (on the y-axis, in thousands) needed to achieve 80% power at the 10^−6^ significance level as a function of “sparsity”, $k_{1}/\sqrt{k}$ (on the x-axis), with *k* and *k*_1_ as defined in the text. It is assumed for these calculations that the *k* SNPs are independent (no linkage disequilibrium), with the minor allele frequency (MAF) sampled from a beta distribution with parameters selected to match allele frequencies from the CEU of the 1000 Genomes Project, *B*(0.14,0.73); the distribution is truncated at 0.01 (so SNPs have MAF < 1%) and only polymorphic SNPs when sequencing 10,000 subjects are selected. Calculations are based on the NCP in Equation (1). OR, odds ratio.

Sparsity of signals and variant annotation: The next term in Formula (1) has to do with the number of associated SNPs relative to investigated SNPs, which we call the sparsity of the signal. Figure 1 shows the impact of sparsity on sample sizes needed to design powerful association studies. For GWAS (*k*_1_ = *k* = 1), the sparsity term is equal to one for associated SNPs. In sequencing studies, it is possible to increase the power by reducing the number of non-associated SNPs (for sets where *k* is large compared to *k*_1_). The annotation of SNPs through functional status, eQTL (expression quantitative trait loci) studies, ENCODE, prior data, *etc.*, will allow more useful definitions for the analyzed sets by excluding SNPs with a low *a priori* likelihood of being associated. This is a fruitful area of current research and one that is implicit in some study designs, such as exome sequencing.

The MAF distribution: The dominant term in the denominator of Formula (1) is given by the mean MAF, so a simple approximation to the third component of NCP is $\sqrt{E_{M}}$. This is the term in NCP that explains most of the difference between sequence association and GWAS. The corresponding term for a single-SNP test with a risk allele frequency of 0.2 is approximately 10 times larger than our 1000 Genomes-based estimate. In order to have comparable power between sequencing and GWAS, this loss would have to be balanced by the other terms (sample size, sparsity and effect size). There is no easy strategy to increase this term in unrelated individuals, but one available route is to shift the study design to families or isolated populations, where alleles which are rare in the larger population are locally common.

Phenotyping/environment: We can also increase power by analyzing datasets with a larger effect size; this corresponds to the last term in Formula (1), (*γ* − 1). This can be done using stratified analysis: by analyzing sub-phenotypes and/or by accounting for environment (when GxE is present). This is a common issue for GWAS and sequencing, and we illustrate the impact of stratification on the effect size using a single SNP as the unit of analysis. Let us assume that *γ_T_* is the mean effect corresponding to the cases in the most at risk strata (with the rest of cases being “controls” with respect to the variants in the set under investigation). Let *α* be the proportion of relevant cases, and let *p* be the control MAF. It follows that in the full set of cases (relevant and irrelevant), the MAF is approximated by *αpγ_T_* +(1−*α*)*p*, and the corresponding effect size is *γ* − 1 ≈ *α*(*γ_T_* − 1). Therefore, if phenotyping or sub-setting by environment allows one to find the relevant cases, analyzing a smaller sample size (of *αn*) leads to an increase of 1/*α* in the fourth term of NCP and to a $1/\sqrt{\alpha }$ overall increase in NCP.

## 3. Prediction Using Rare and Novel SNPs: A Different Winner's Curse

Aside from association discovery, one of the major goals of GWAS is to estimate the effect sizes of SNPs on traits, which can be used for the prediction of unrealized phenotypes on newly sequenced individuals. For example, SNP genotyping platforms have recently been used for risk and pharmacogenomic prediction by several companies, such as 23andMe, Life Technologies, and Pathway Genomics. Prediction using SNPs discovered in a sequencing study can be performed analogously to GWAS, as long as adequate data has been gathered. One major difference between GWAS and sequencing is that newly-sequenced individuals will regularly carry novel SNPs in disease-associated genes, and most discovered SNPs will have too little information for accurate per-SNP estimates [13,14]. Quantitative estimates of personal risk based on sequencing association studies will therefore require an evidence-based estimate of the effect of previously unobserved and seldomly observed rare SNPs. Given that mutations in the gene in question have already been associated with disease and that harmful SNPs are thought to be more likely to be rare [15,16,17,18,19,20], ignoring these SNPs (setting the effect to zero) is unlikely to be accurate. Naively, we could estimate a “rare SNP effect” based on the rare SNPs observed in previous sequencing studies and apply that estimate to new SNPs and known rare SNPs alike. We illustrate two problems with no analog in GWAS that occur when rare SNPs are lumped into a super-SNP for estimation or prediction. The major results are that: (1) rare alleles in a sequencing study can cumulatively have a substantial per-allele OR, which depends on disease prevalence, even if log odds-ratios (lORs) are centered at zero; (2) the prediction of new samples based on that OR is substantially inaccurate.

First, unlike GWAS, prediction with new SNPs depends non-trivially on the variability of rare SNP effects. With GWAS, previous data will give the investigator an estimate of the effect of each SNP; a plug-in prediction can be formed using these estimates: *logit*(*Ŷ_i_*) = *G_i_β̂* + *α*, where *logit* is the logistic function, *Ŷ_i_* is the predicted probability for person *i*, *G_i_* is the vector of genotypes for that person, *α* is an intercept, which depends on disease frequency, and *β* are SNP lORs. The naive plug-in prediction is not quite correct due to the uncertainty in SNP effects; however, the inaccuracy with GWAS-based estimates tends to be negligible for reasons discussed below. In contrast, the effect of a rare SNP in an associated gene is not precisely known, and the impact of that uncertainty on prediction is substantial. For example, even if SNPs in an associated gene are as likely to be risk-decreasing as risk-increasing, the correct prediction in the context of a rare disease for a newly sequenced individual is that carrying a novel SNP increases their odds of being affected. Qualitatively, the uncertainty in SNP effects makes one less confident in the plug-in estimate and pushes the best estimate from the raw prevalence closer to 50:50. To give a numerical example, if the population of lORs for novel SNPs is Gaussian, with a mean of zero and standard deviation of one, and the disease frequency is 1%, then the marginal OR for carrying an allele (*versus* no minor alleles) is 1.9.

This is a well-known phenomenon from the literature comparing marginal and conditional random effects [21,22,23]; derivation of the effect size and additional explanation is offered in the Appendix. A useful formula for the risk associated with carrying new SNPs can be derived under the assumption that their lORs are Gaussian distributed with mean *μ* and standard deviation *σ* along with standard logistic regression assumptions. Define c as a constant related to the disease prevalence (the threshold in the Appendix $c=-\text{log}\frac{p}{1-p}\approx -\text{log}(p))$ and *g_i_* as the number of alleles in that individual, then:

(2) $$\text{logit}(\mathrm{Pr}\{Y_{i}=1|g_{i},\mu ,\sigma \})\approx \frac{-c+\mu g_{i}}{\sqrt{1+\nu^{2}\sigma^{2}g_{i}}}$$

where *ν* ≈ 0.625. When the mean lOR is zero and the standard deviation is not large (it is almost assuredly less than one in areas without overwhelming evidence for linkage), the lOR for having an SNP (*versus* no SNPs) is approximately $\frac{c\nu^{2}\sigma^{2}}{2\sqrt{1+\nu^{2}\sigma^{2}}}$, which increases sharply with the standard deviation of lORs and scales with the negative log of disease prevalence. This is the lOR that we estimate when regressing the outcome on the number of SNPs carried and that we would use for prediction, absent other information about the effects of particular SNPs.

A related result occurs for GWAS-based plug-in estimates, the details of which depend on the choice of statistical estimators used for effect estimates and the pattern of linkage disequilibrium. For any estimate of an SNP's lOR for which a central limit theorem applies, *σ*^2^ in Equation (2) can be replaced with the square of the standard error of the estimate, which will usually scale as the inverse of the sample size and the MAF Given the relatively low cost of GWAS data acquisition and the need to overcome the burden of genome-wide multiple testing, we are accustomed to gathering enough data for precise estimates. For example, the expected standard error of a lOR of zero with a MAF of 0.3 and 3000 cases and 3000 controls is 0.06. If the standard deviation of the population of novel SNP lORs is 0.5, then the marginal lOR for a new SNP is 70 times bigger than the previously observed SNP.

The above effect is observable regardless of the sample size and MAF of SNPs used in the calculation. One might expect that since case control-based estimates of ORs are consistent for prospective associations, that this effect would be corrected by empirically estimating the per-allele OR and using that for future data; however, there is a unique twist for the group of SNPs with MAFs, such that they are reasonably likely to be monomorphic in the original study. The observed lOR for all rare SNPs together does estimate the marginal effect of future rare SNPs, but that prediction breaks down when stratified by whether or not the SNP was observed as polymorphic in the case-control study. Case-control designs are somewhat more efficient for discovering rare risk-increasing SNPs compared to risk-decreasing SNPs [20,24,25,26,27], so as a group, previously observed SNPs are more harmful in future samples than newly observed SNPs.

**Figure 2:**
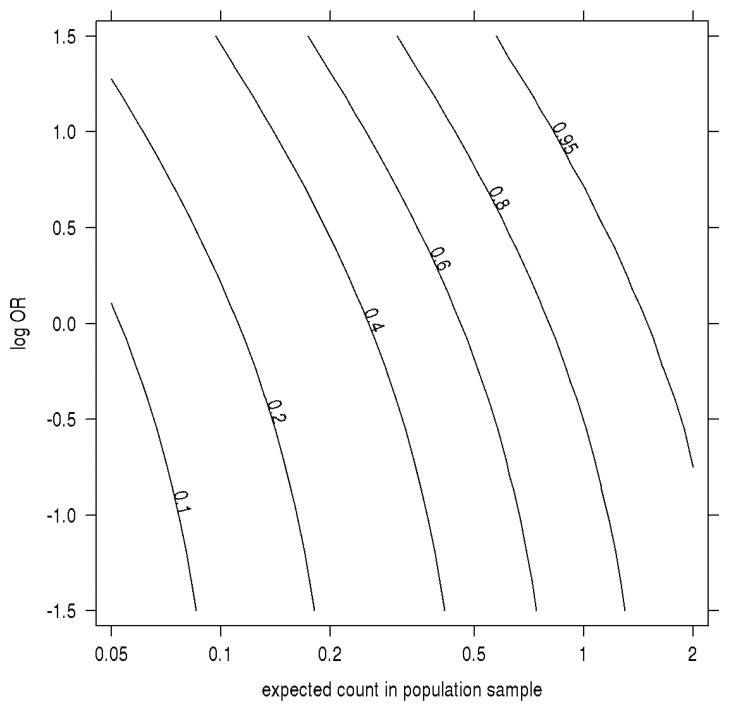
Sampling probability by MAF, log odds-ratio. The contour plot has on the x-axis the allelic expected count in a population sample the same size as the control group (sample sizes times MAF) and, on the y-axis, the log-odds ratio. Contours are the absolute probability of being sampled in a case-control study of 100 cases and 100 controls when prevalence equals 1%.

In Figure 2, we plot how the probability of an SNP being discovered (the minor allele is observed in at least one participant) in a case-control study depends on both the OR and the MAF when the MAF is low compared to the sample size. The absolute probability of a SNP appearing at least once in the study increases markedly with OR in this range of MAF. Intuitively, compared to a population sample, a risk-increasing SNP's greater frequency among cases more than makes up for the decline in its frequency among controls. As a result, the observed odds of a rare SNP appearing in a case are inflated, and the finite pool of remaining SNPs at that MAF contains a preponderance of protective and small effects.

**Figure 3:**
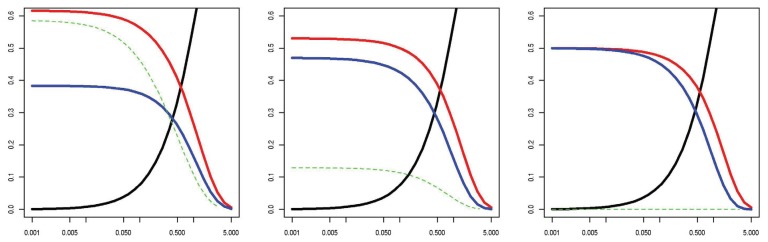
Observed data probabilities by MAF. X-axis N*·*MAF. The y-axis shows the probability of each special data type conditional on the SNP being polymorphic: occurring only in cases (red), once in controls and zero times in cases (blue) and all other (black). Green = expected log-odds-ratio (OR) of sampled SNPs (same numeric scale). The log-ORs are assumed to be distributed left: *N*(0, 1); center: *N*(0, 0.5^2^); right: *N*(0, 0.25^2^); other settings are as in Figure 2.

This is similar to the first problem discussed above, except that we have selectively observed SNPs based on their true odds ratio. Figure 3 shows that when the lORs of SNPs in a gene are assumed to come from a population with high variance, the odds of an SNP appearing only in cases and the expected lOR of observed SNPs varies with MAF and substantially favors an increase in risk. This is not a Bayesian argument; it relies only on the rate at which SNPs appear in the sample, even for fixed SNP effects. To give a numerical example, with a sample size of 100, a prevalence of 5%, 125 SNPs with a MAF of 0.002 and lORs drawn from a standard normal, an average of 36% of the SNPs are discovered in the original sequencing study with an average per-allele estimated lOR of 0.33. In a new replication or prediction sample, the average per-allele lOR based on previously discovered SNPs is 0.66, but the per-allele lOR of new SNPs is only 0.05. The numerical result depends heavily on a number of parameters; we have deferred a detailed exploration of the phenomenon to another work [28]. The longer report is available for download, and re-demonstration of the importance of each factor is beyond the scope of this paper. However, there are three notable features to which we wish to briefly draw attention: (1) the tail behavior of SNP lORs is very influential; large ORs enrich even rare SNPs into the population of cases; (2) the effect occurs at a MAF around the minimum observable in a study; regardless of the size of the original dataset observed, rare SNPs are unrepresentative of future rare SNPs; (3) the effect vanishes under the null hypothesis that no SNPs in a gene are associated.

## 4. Implicit and Explicit Models in Association Studies

In contrast to GWAS, genetics practitioners with sequencing data are currently faced with a dizzying selection of methods to test for an association between genotype and phenotype, each of which has tuning parameters. In GWAS, a simple allelic test is the overwhelmingly most commonly used test. The additive allelic model performs well regardless of the true risk model when linkage disequilibrium between a tested marker and causal allele is imperfect [29]. While some authors have suggested GWAS schemes that incorporate prior knowledge and more complex risk models through explicit Bayesian calculations or alpha-spending procedures (see also Section 5), the dominant technique in the literature is to report SNP-level evidence and the allelic effect from a particular dataset. While the commonly used methods make usual regression-type assumptions about the distribution of the trait, the effects of confounders and covariates and the measurement error of SNPs, they make minimal assumptions about the effects of other SNPs or how effect size varies with SNP- or gene-level features.

Because of the small amount of information at each rare SNP, all sequencing association tests of which we are aware pool information in some way across SNPs, which are regarded as belonging to a unit (gene) or being “similar,” and some pool information across genes that are “similar.” These techniques have tuning parameters appropriate under a particular alternative hypothesis and that may suffer a substantial loss of power under other alternatives. A full comparison of proposed tests for sequencing data is beyond the scope of this article; however, we will discuss a few of the most common tests. In this section, we will discuss the role and meaning of some of these tuning parameters. Ignoring these tuning parameters as if the investigator were still using relatively assumption-free GWAS techniques is unlikely to work well, and the importance of these analytic decisions represents a substantial divergence from GWAS.

One extreme of this approach is to try to swap tuning parameters for explicit models and assumptions. We have advocated multi-level modeling of effect sizes or lORs using SNP- and gene-level features as predictors, with stated assumptions, such as the functional form of associations, the linearity and additivity of associations, distributional requirements and exchangeability between SNPs, where required [30].

**Figure 4:**
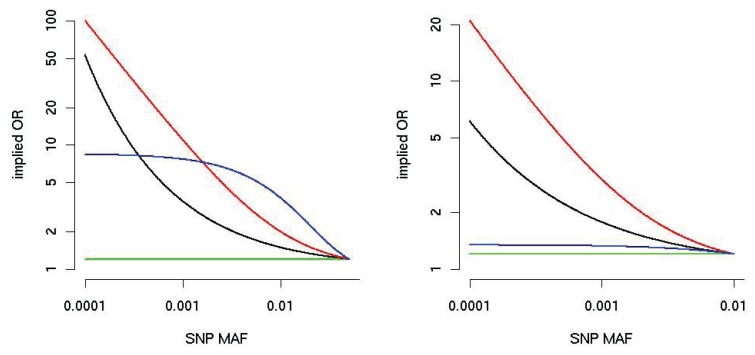
Implied alternative OR (on the y-axis, logarithmic scale) as a function of MAF (x-axis) for three burden weighting schemes. The black line corresponds to the Madsen– Browning weight [10]; the red line corresponds to the attributable risk weight [41], and the blue line corresponds to the default in SKAT, Beta(25,1) [42]; the green line is for equal weights. For the **left** panel, the MAF is truncated at 5%, and for the **right** panel at 1%. We assume that the OR of the SNP with the largest MAF is 1.2.

However, most proposed tests are not model-based summaries. In some cases, we can gain insight into these tests by constructing a map from the tuning parameters to a genetic model, which would imply those as optimal in some way. For example, *C*(*α*) and diagonal kernel SKAT [5] can be derived from an explicit model with weights on the *j* — *th* SNP, $w_{j}\propto E[lOR_{j}^{2}]$ [31], and therefore, any scheme of weights as a function of MAF can be understood in terms of the implied variance of SNP effects. The addition of correlation structures to SNP effects also follows a simple model-based logic; for example, if effects are expected to substantially go the same direction (such as a group of loss-of-function SNPs), one can balance between the burden-type and variance-component-type test [5]. Similarly, several authors [32,33] have pointed out that optimal weights for burden-type tests are proportional to per-SNP lORs. Figure 4 shows curves for three MAF-depended weights proposed in the literature [34]. Notably, the implied lOR curve depends heavily on the upper limit of MAF included in the pooling procedure.

However, the tuning parameters of some proposed tests are more challenging. For example, SKAT with the Gaussian kernel does not map to a meaningful model of SNP effects, but is suspected to work reasonably well under several alternatives and detects some non-linear effects and epistatic interactions [35,36,37]. The kernel itself is a tuning parameter for SKAT and related methods; kernel-based techniques are sensitive to the choice of kernel and, aside from a few special cases [35,38], are difficult to choose between *a priori*. Additionally, while most kernels can up- or down-weight SNPs, transforming prior information into a calibrated SNP “similarity” or “distance” measure is a task for which we have little guidance. While SKAT and related tests can be motivated by a simple variance components model, they are not automatically robust to non-Gaussian SNP effects, such as a mixture of causal and non-causal alleles [39,40]. This is not to suggest that the many variants of kernel-based tests are poorly applied tools or that they perform poorly compared to other tests, just to highlight the fundamental difficulty of interpreting and guiding the key analytic decision.

There are numerous specific deviations from linear Gaussian SNP effects, which hypothetically should influence the tuning parameter selection. In the implicit model tests described above, these issues are difficult to address in planning and power analysis. When considering sequencing data as potential negative evidence in replication studies, each has to be explored on a case-by-case basis. Explicit-model methods have the advantage of facilitating graphical model checks (for an example, see [30]), posterior-predictive diagnostics [43] and prior-data conflict summaries [44,45]. The price of these checks is a relatively high computational burden, stricter distributional assumptions, additional investigator effort eliciting the model (and suitable priors for Bayesian methods [46]) and an unclear definition of a “good enough” model.

## 5. On Multiplicity

The common strategy used in GWAS for ranking and follow-up of new discoveries is to focus on the SNPs with the most significant *p*-values. As we discussed in Section 2, association tests of common single SNPs yield *p*-values that reflect a combination of sample size, effect size and MAF; however, for the range of MAFs in GWAS, rankings based on *p*-values correlate well with those based on effect size. Adjustment for multiple testing is usually done independent of any information on SNPs, and in a Bayesian framework, this corresponds to an equal prior probability of each SNP being associated. There have been approaches developed to incorporate prior knowledge, such as stratified false discovery control [47] and weighted Bonferroni criteria [48]. Although there are several sources of information for these procedures in GWAS (such as effect on expression, effect on related phenotypes, position relative to gene elements, MAF), formal methods have not been used extensively for several reasons. In general, the available information is difficult to translate to the right scale, and there is low prior confidence that information on tag-SNPs is useful, since the causal SNP is unobserved.

Sequencing-based association studies are even more challenging, because there is more variability in the units of analysis than in GWAS. Gene units vary enormously in the number of SNPs, linkage disequilibrium (LD) pattern, the plausible ratio of causal SNPs, MAF spectrum and annotations. For example, what is more likely to be associated, a gene with two non-synonymous SNPs or a gene with ten non-synonymous SNPs? A gene with ten singletons (variants with only one observed copy of the non-reference allele) *versus* a gene with 10 total minor alleles with varying MAF? A set of ten non-synonymous SNPs *versus* a set of ten intronic SNPs? Furthermore the calculation of optimal weights will include an interplay of subject matter knowledge (e.g., assumptions on the effect sizes for different annotations) and the choice of statistical methods (e.g., some methods will accommodate signal sparsity well).

The complicated assessment of prior probabilities for a set of SNPs is one of the issues in using *p*-values for ranking genes and for deciding on efficient follow-up studies. *p*-values might be a poor proxy for the probability of replication, especially when the signals come from the very rare alleles that might not appear in the subjects used for replication. *p*-values contain little information on strategies for functional validation, because they do not inform on the best variants to be investigated. Relying only on *p*-values for decision-making has a bigger impact in sequencing studies than in GWAS, and we hope that developing better and more diverse measures of significance will become a more active area of research.

## 6. Discussion

Many people have been surprised by the lack of substantial findings from the recent studies on rare variants performed with whole-genome or whole-exome sequencing and from platforms, such as the exome chip. The reality is that for complex traits, there was little prior evidence in favor of genetic models that would give such studies high power (with multiple rare variants with a large effect per unit of study). The whole literature of the recent past, which is too extensive to be cited here, on investigating low frequency variants using imputation from population-based sequencing shows that large effect SNPs are uncommon for the diseases where they exist. This advocates for the development of more efficient strategies than the brute force sequencing of large, poorly phenotyped cohorts. The detailed annotation of variants should improve the sparsity of signals in the units of analysis, and careful phenotyping and incorporation of environmental factors should lead to the discovery of larger effects.

Much of the analytical effort on the association with sequencing data has been put into the development of novel testing tools. We argue in this paper that it is equally important to focus on other aspects of the process, from the design of the study to the interpretation of results. Furthermore, hypothesis tests and multiplicity adjustments should fit into the paradigm of a careful design that we set out above; model-based tests should incorporate the complexity that we expect without resorting to black boxes or poorly characterized weights. We should also be on guard for excessive parsimony; lumping together rare SNPs into a super-SNP creates a variable with properties that depend on the sampling scheme, minor allele frequency distribution and effects on phenotype distribution in complex ways.

## Figures and Tables

**Figure A1:**
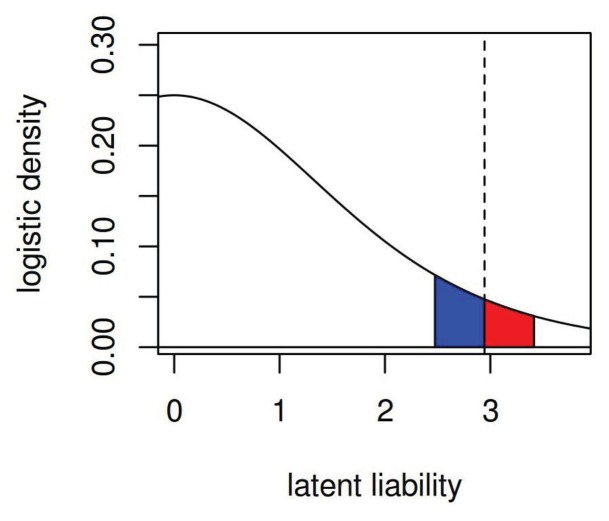
Density of latent trait before SNP effects. The dotted line indicates the case threshold. The blue area corresponds to controls that become cases if possessing an SNP with OR = 1.6. The red area indicates cases that become controls if possessing an SNP with lOR = 1/1.6.

## Appendix

### A. The Derivation of the Power Formula

The following assumptions are used for the calculation of power: (1) there are *n* cases and *m* controls; (2) the association test is performed on a set of *k* SNPs, out of which, *k*_1_ re associated, and the calculations are done conditional on *k* and *k*_1_, ignoring the variability in those numbers that is associated with sequencing; (3) MAFs are sampled from a distribution with mean *E_M_* and variance *V_M_*; (4) for simplicity, we assume that the effect sizes of the associated SNPs are independent of MAF; (5) the SNPs are in linkage equilibrium; (6) all associations are with the rare allele; and (7) the effect sizes are sampled from a distribution with a mean odds ration equal to *γ*.

The association method used for illustration is the “burden” test, where, for each individual, we calculate a score based on the genotypes for the *k* SNPs. Let *G_j_* denote the number of rare alleles at the *j*-th SNP, and let *w_j_* be a fixed prespecified weight (it does not depend on the observed data; this is needed to simplify the analytical calculations). For each subject, we calculate:

$$S=\sum_{j=1}^{k}w_{j}G_{j}$$

then correlate this with the trait for the detection of association. For case-control studies, this can be done using a two-sample *t*-test. Note that power for a two sample normal test is governed by the non-centrality parameter,

$$\sqrt{\frac{nm}{n+m}}\frac{\mu_{1}-\mu_{2}}{\sigma }$$

with classical notation (and assuming equal variance). Note that for large *k* and *n*, the burden test will be close to a two sample normal test, and the mean and variance of the scores are the key determinants of power. The approximation is fairly accurate, even for small *k*, as long as *n* remains large. If we denote with *P_j_* the MAF for the *j*-th SNP, we have that:

$$\text{E}(S)=\text{E}[\text{E}(S|P)]=\text{E}\left(\sum_{j=1}^{k}w_{j}2P_{j}\right)=\sum_{j=1}^{k}2w_{j}E_{M}=2\bar{w}kE_{M}$$

where *w̄* is the average weight. Similarly,

$$\begin{array}{c} \text{Var}(S)=\text{Var}[\text{E}(S|P)]+\text{E}[\text{Var}(S|P)]=\text{Var}\left(2\overset{k}{\underset{j=1}{\text{∑}}}w_{j}P_{j}\right)+\text{E}\left(2\overset{k}{\underset{j=1}{\text{∑}}}w_{j}^{2}P_{j}(1-P_{j})\right)=\\ 4V_{M}\overset{k}{\underset{j=1}{\text{∑}}}w_{j}^{2}+2\left(\overset{k}{\underset{j=1}{\text{∑}}}w_{j}^{2}\right)(E_{M}-V_{M}-E_{M}^{2})=2k\bar{w^{2}}[V_{M}+E_{M}-E_{M}^{2}] \end{array}$$

In the case of equal weights (*w_j_* = 1),

$$\begin{array}{cc} \text{E}(S)=2kE_{M}, & \text{Var}(S)=2k(V_{M}+E_{M}-E_{M}^{2}) \end{array}$$

For a rare associated SNP, its MAF is approximated by the product of the MAF in controls and the odds ratio. Because MAF and the odds ratios are independent (Assumption 4), we obtain that the mean MAF is approximated by *E_M_γ*. This leads to the following mean score in cases (assuming equal weights),

$$\text{E}(S)\approx 2(k-k_{1})E_{M}+2k_{1}E_{M}\gamma =2kE_{M}+2k_{1}E_{M}(\gamma -1)$$

One can similarly derive a formula for Var(*S*) in the cases.

Assuming that the variances of the scores are not greatly different in cases and controls (valid with mild assumptions), the non-centrality parameter is approximated by:

$$\sqrt{\frac{2nm}{n+m}}\frac{k_{1}}{\sqrt{k}}\frac{E_{M}}{\sqrt{V_{M}+E_{M}-E_{M}^{2}}}(\gamma -1)$$

### B. Marginal Effects with Uncertainty

The marginal effect of a SNP whose true log odds ratio comes from a known distribution is easy to quantitatively analyze when using a model of the binary disease outcome as a dichotomized latent liability plus an SNP effect. That is, one can recast a traditional logistic regression model as a model where each individual has an unobserved quantitative trait, and individuals whose quantitative trait is greater than some threshold demonstrate a positive binary trait. Effects of covariates (such as SNPs) add or subtract to the unobserved quantitative trait; when the liability has a logistic distribution (slightly heavier tailed than a Gaussian distribution), the effects on the latent scale are the same as lORs. Consider a logistic model shown in Figure A1; the plotted curve is the density of the latent liability and the dotted line the threshold above which individuals are affected by disease and below which they are unaffected if the base rate of the disease is 5%. The area under the curve to the right of the threshold are affected individuals and to the left of the threshold unaffected. The blue area under the curve is the fraction of individuals in the population who, with a moderately risk-increasing SNP, would cross the liability threshold and become affected by the disease. The smaller red area is the individuals who, with a risk-decreasing SNP of the same magnitude, lOR would cross the liability threshold in the other direction and cease to be affected.

When *G_i_* is a vector of genotypes for person *i*, *β* are SNP lORs Gaussian distributed with mean *μ* and standard deviation *σ*, c is the threshold above, *I*() the indicator function and *X_i_* the latent liability, then we can write:

(A1) $$Y_{i}|g_{i},\mu ,\sigma =I(X_{i}+G_{i}\beta >c)$$

One can approximate a logistic variable by a Gaussian scaled by 1.6, yielding:

(A2) $$Y_{i}|g_{i},\mu ,\sigma =I(Z_{i}\sqrt{1.6^{2}+\sigma^{2}g_{i}}+\mu g_{i}>c)$$

for *Z_i_*, a standard normal. One can then re-apply the normal-logistic approximation:

(A3) $$Y_{i}|g_{i},\mu ,\sigma =I(X_{i}^{*}+\frac{\mu g_{i}-c}{\sqrt{1+\sigma^{2}g_{i}/1.6^{2}}}>0)$$

where *X** is again logistic distributed, returning to a usual form for logistic regression.
